# Pyramiding of transgenic *Pm3* alleles in wheat results in improved powdery mildew resistance in the field

**DOI:** 10.1007/s00122-017-3043-9

**Published:** 2018-01-04

**Authors:** Teresa Koller, Susanne Brunner, Gerhard Herren, Severine Hurni, Beat Keller

**Affiliations:** 1 Department of Plant and Microbial Biology, University of Zurich, Zollikerstrasse 107, 8008 Zurich, Switzerland; 2 Agroscope, Reckenholzstrasse 191, 8046 Zurich, Switzerland

## Abstract

**Key message:**

The combined effects of enhanced total transgene expression level and allele-specificity combination in transgenic allele-pyramided Pm3 wheat lines result in improved powdery mildew field resistance without negative pleiotropic effects.

**Abstract:**

Allelic *Pm3* resistance genes of wheat confer race-specific resistance to powdery mildew (*Blumeria graminis* f. sp. *tritici*, *Bgt*) and encode nucleotide-binding domain, leucine-rich repeat (NLR) receptors. Transgenic wheat lines overexpressing alleles *Pm3a, b, c, d, f,* and *g* have previously been generated by transformation of cultivar Bobwhite and tested in field trials, revealing varying degrees of powdery mildew resistance conferred by the transgenes. Here, we tested four transgenic lines each carrying two pyramided *Pm3* alleles, which were generated by crossbreeding of lines transformed with single *Pm3* alleles. All four allele-pyramided lines showed strongly improved powdery mildew resistance in the field compared to their parental lines. The improved resistance results from the two effects of enhanced total transgene expression levels and allele-specificity combinations. In contrast to leaf segment tests on greenhouse-grown seedlings, no allelic suppression was observed in the field. Plant development and yield scores of the pyramided lines were similar to the mean scores of the corresponding parental lines, and thus, the allele pyramiding did not cause any negative effects. On the contrary, in pyramided line, Pm3b × Pm3f normal plant development was restored compared to the delayed development and reduced seed set of parental line Pm3f. Allele-specific RT qPCR revealed additive transgene expression levels of the two *Pm3* alleles in the pyramided lines. A positive correlation between total transgene expression level and powdery mildew field resistance was observed. In summary, allele pyramiding of *Pm3* transgenes proved to be successful in enhancing powdery mildew field resistance.

## Introduction

In agricultural practice, two important strategies to control crop diseases are the use of pesticides and the use of disease-resistant cultivars. To generate resistant cultivars, a common approach is the introgression of resistance genes found in the gene pool of the crop species of interest by multiple steps of backcrossing (Visscher et al. 1996; Hillel et al. 1990; Tanksley and Nelson 1996). A faster and more precise method of introgression is the genetic transformation of high yielding crop cultivars with resistance genes of interest (Narusaka et al. 2013; Rodriguez-Moreno et al. 2017; Lacombe et al. 2010; Mondal et al. 2016). Resistance gene *Pm3* from wheat confers resistance to powdery mildew (*Blumeria graminis* f. sp. *tritici*, *Bgt*). So far, 17 functional *Pm3* alleles have been identified (Yahiaoui et al. 2004, 2006; Srichumpa et al. 2005; Bhullar et al. 2009, 2010). The *Pm3* alleles encode nucleotide-binding domain; leucine-rich repeat (NLR) type of receptors which are able to recognize effectors from *Bgt* and subsequently trigger a hypersensitive immune response. The interaction of wheat and *Bgt* is complex, involving many *Bgt* effectors, some of which act as avirulence factors as in case of effector AvrPm3^a2f2^ (Bourras et al. 2015). AvrPm3^a2f2^ has been identified as being recognized by the two Pm3 variants Pm3a and Pm3f (Bourras et al. 2015). Recently, effector SvrPm3^a1f1^, which acts as a suppressor of recognition, has been identified, disclosing another layer of complexity of the wheat–*Bgt* interaction (Bourras et al. 2015, 2016; Parlange et al. 2015). Wheat lines carrying *Pm3* transgenes have been generated by biolistic transformation of spring wheat cultivar Bobwhite (Brunner et al. 2011, 2012) which does not carry an endogenous *Pm3* allele. Four transgenic wheat lines carrying the allele *Pm3b* at different loci in the genome were generated and tested in field trials. The Pm3b lines were more powdery mildew resistant than their corresponding sister lines (null segregants of the transgene) (Brunner et al. 2011). In addition to the Pm3b lines, transgenic wheat lines carrying alleles *Pm3a*, *Pm3c*, *Pm3d*, *Pm3f*, and *Pm3g* were generated and two lines for alleles *Pm3a*, *Pm3c*, and *Pm3f* and one line for alleles *Pm3d* and *Pm3g* and all corresponding sister lines were tested in the field, together with three line mixtures consisting of 1:1 seed mixtures of lines Pm3a + Pm3b, Pm3a + Pm3d, and Pm3b + Pm3d. The transgenic lines showed increased powdery mildew resistance compared to their sister lines and the multilines were more powdery mildew resistant than the transgenic lines in pure stands (Brunner et al. 2012).

Several resistance genes or several alleles of the same resistance gene can be combined, or as often called “pyramided”, in one plant genotype to further improve resistance in terms of durability and spectrum. If several pyramided resistance genes are present in the plant encoding receptors which are able to detect different effectors of a pathogen, the pathogen has to modify multiple effectors to not trigger an immune response in the plant. If several pyramided alleles of a resistance gene are present in the same plant and these alleles encode receptors, which are able to detect different effectors present in different isolates of the pathogen population, the plant is able to detect a wider range of the pathogen population. Thus, pyramiding of resistance genes and resistance alleles has the potential to prevent or delay the development of boom-and-bust cycles commonly observed when only single- resistance genes are deployed (McDonald and Linde 2002; Zhan et al. 2015; Burdon et al. 2014; Delmotte et al. 2016). Pyramiding of resistance genes can be achieved by classical crossbreeding or by genetic engineering. Pyramiding of several alleles of the same resistance gene can only be achieved by genetic engineering. A prominent example of successful resistance gene pyramiding by genetic engineering is the combination of late blight-resistance genes in potato (Zhu et al. 2012; Jo et al. 2014). An example of gene pyramiding by genetic engineering for improved insect resistance is the combination of transgenes encoding *Bacillus thuringiensis* (*Bt*) toxins in maize and rice (reviewed in Liu et al. 2016). Stirnweis et al. (2014) generated *Pm3* allele-pyramided lines by crossbreeding transgenic lines carrying single *Pm3* alleles. They generated the four double homozygous lines Pm3a × Pm3b, Pm3a × Pm3d, Pm3b × Pm3d, and Pm3b × Pm3f. They tested the powdery mildew resistance of the pyramided lines in infection tests on leaf segments in the laboratory. Surprisingly, for some but not all *Bgt* isolates, a resistance suppression was observed, namely Pm3 variants Pm3b and Pm3c suppressed the function of the two Pm3 variants, Pm3a and Pm3f (Stirnweis et al. 2014). Pm3b suppressed the triggering of a hypersensitive response of autoactivated Pm3f in *Nicotiana benthamiana* assays and the authors were able to pinpoint the LRR domain as the domain conferring suppression (Stirnweis et al. 2014). Furthermore, in co-immunoprecipitation assays, the authors showed an interaction of Pm3b and Pm3f (Stirnweis et al. 2014). The objective of this study was to test the pyramided lines Pm3a × Pm3b, Pm3a × Pm3d, Pm3b × Pm3d, and Pm3b × Pm3f, all in the background of cultivar Bobwhite, in the field, to assess powdery mildew field resistance and pleiotropic effects of the pyramided transgenes on plant development and yield.

## Materials and methods

### Transgenic wheat lines

Transgenic wheat lines in the genetic background of spring wheat cultivar Bobwhite SH 98 26 carrying alleles *Pm3a, b, d,* or *f* and their corresponding sister lines were previously generated and described by Brunner et al. (2011, 2012) (named Pm3a#1, Pm3b#1, Pm3d#1, Pm3f#1, Sa#1, Sb#1, Sd#1, and Sf#1). Pyramided lines Pm3a × Pm3b, Pm3a × Pm3d, Pm3b × Pm3d, and Pm3b × Pm3f were previously generated and described by Stirnweis et al. (2014). The following seed generations were used in 2015 field trial: **T4** (Pm3b, sister line Pm3f), **T5** (Pm3a, Pm3d, Pm3f), **F5** (Pm3a × Pm3d, Pm3b × Pm3d), and **F6** (Pm3a × Pm3b, Pm3b × Pm3f). The following seed generations were used in 2016 field trial: **T4** (sister line Pm3f), **T5** (Pm3b), **T6** (Pm3a, Pm3d, Pm3f), **F6** (Pm3a × Pm3d, Pm3b × Pm3d), and **F7** (Pm3a × Pm3b, Pm3b × Pm3f). Seeds of the transgenic lines and the corresponding sister line were produced in the field in 2014 and 2015, respectively.

### Field trial

Field trials were carried out at the “Protected Site” of the Swiss centre of excellence for agricultural research Agroscope in Zurich—Reckenholz during field seasons 2015 and 2016. The “Protected Site” is a site for field trials with genetically modified plants (http://www.protectedsite.ch; Romeis et al. 2013). The site is protected to prevent vandalism. Microplots in both years had a size of 1.32 m × 1.0 m. Five plots per line or cultivar were grown in a randomized complete block design. In 2015, yield plots had a size of 6.0 m × 1.5 m and in 2016 a size of 5.8 m × 1.5 m. Four plots per line were grown in a randomized complete block design. We determined germination rates and thousand kernel weights of each line and cultivar and sowed 400 viable kernels per m^2^. Seeds were treated with 2 ml/kg Celest Trio (Syngenta) and 1.5 ml/kg Smaragd (Bayer). Each yield plot was bordered on all sides by plots of powdery mildew-resistant triticale cultivar Trado, leading to a chessboard-like design, and in addition, 1.32 m-wide transverse rows of triticale between the short sides of all neighboring plots to avoid direct corner-to-corner contact between the test plots. Micro and yield plots were flanked by infection rows consisting of the powdery mildew susceptible wheat breeding line FAL94632 and cultivar Kanzler. Pots with susceptible wheat plants pre-infected in the greenhouse with *Bgt* isolate 96224 were planted into the infection rows. The test area was surrounded by a 3 m-wide border crop consisting of triticale cultivar Trado. Application of fertilizer and insecticides was performed according to the standard Swiss agricultural practices. No fungicides were applied. Powdery mildew disease symptoms and plant development phenotypes were scored as previously described by Brunner et al. (2011, 2012).

### *Pm3* transgene expression analyses by RT qPCR

Leaf samples were collected in 2015 and 2016 from plants grown in microplots. Segments of the fully developed flag leaves from three plants per plot were pooled in one tube and immediately frozen on dry ice and stored at − 80 °C. Expression of *Pm3* was quantified in a reverse transcription, quantitative real-time PCR (RT qPCR) assay, using a CFX384 Real-Time System C1000TM Thermal cycler (Bio-Rad). RNA extraction was performed as described by Hurni et al. (2015), and first-strand cDNA was synthesized from 0.5 μg RNA, using 1/2 reaction of the iScript Advanced cDNA Kit (172-5038, Bio-Rad). RT qPCR was performed with 2.4 µl of 20-fold-diluted cDNA in 6 µl and technical triplicates. Specificities of amplicons, RT-minus control check, and efficiency calculation were performed as described in Hurni et al. (2015). Relative quantities were calculated and normalized to the reference gene Ta.6863 revealing the calibrated normalized relative quantities (CNRQ) values, using the program qbase+ V 3.0 (Biogazelle).

## Results

### Field trials of pyramided lines and evaluation of the *Bgt* population present in the field

**Fig. 1:**
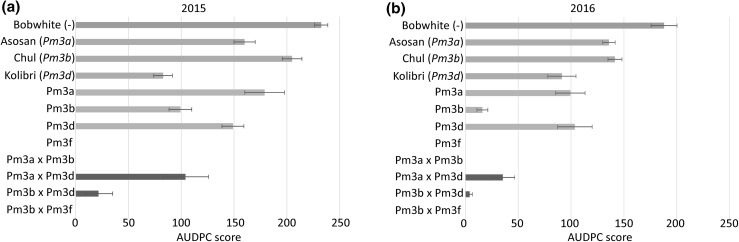
Powdery mildew infection of field grown transgenic pyramided lines (dark green), transgenic parental lines (light green), cultivars carrying endogenous *Pm3* alleles (yellow), and non-transformed Bobwhite (grey). Means of area under disease progress curve (AUDPC) scores were calculated from five independent replicates (five microplots) per line grown in field season 2015 (**a**) and in field season 2016 (**b**). Standard errors of five replicates are indicated (color figure online)

In the field trials, we tested the four pyramided lines Pm3a × Pm3b, Pm3a × Pm3d, Pm3b × Pm3d, and Pm3b × Pm3f, their parental lines Pm3a, Pm3b, Pm3d and Pm3f, and the sister lines of the parental lines at a field site for trials with genetically modified plants in Switzerland. The sister lines are null segregants of the transgene, meaning that no *Pm3* alleles are present. We included in the field trial non-transformed Bobwhite and spring wheat cultivars Asosan, Chul, and Kolibri, which carry the endogenous alleles *Pm3a*, *Pm3b,* and *Pm3d*, respectively. Line mixtures Pm3a + Pm3b, Pm3a + Pm3d, Pm3b + Pm3d, and Pm3b + Pm3f were included in the yield trial for performance comparison of the pyramided line and the line mixture. To increase disease pressure, we planted spreader rows between each line of plots consisting of powdery mildew susceptible wheat plants infected with *Bgt* isolate 96224. This isolate was originally collected in Switzerland and since then cultivated in the laboratory. It was chosen for field infection, because previously conducted leaf segment tests in the laboratory showed avirulence of the isolate on wheat lines carrying any of the alleles *Pm3a, Pm3b, Pm3d,* or *Pm3f* (Brunner et al. 2010, 2011, 2012). We scored powdery mildew infection starting from the time point of onset of the disease, which occurred around 2 months after sowing and subsequently calculated the area under disease progress curve (AUDPC) score for each line. In 2015, Chul (*Pm3b*) (AUDPC score 205) was almost as highly infected as non-transformed Bobwhite (AUDPC score 233), Asosan (*Pm3a*) had an AUDPC score of 160, thus was slightly resistant, and Kolibri (*Pm3d*) with an AUDPC score of 83 was the least infected of the cultivars carrying an endogenous *Pm3* allele (Fig. 1a). In 2016, disease severity was weaker compared to 2015 probably due to the weather conditions. However, the infection pattern in 2016 was very similar: non-transformed Bobwhite was the most infected cultivar with an AUDPC score of 188 followed by Asosan and Chul with similar scores of 136 and 142, respectively, and Kolibri with a score of 91 (Fig. 1b). In summary, in both years, cultivars Asosan (*Pm3a*) and Chul (*Pm3b*) were almost as infected as non-transformed Bobwhite, and cultivar Kolibri (*Pm3d*) showed a medium level of powdery mildew resistance (Fig. 1). Thus, in both years, *Bgt* strains virulent on alleles *Pm3a*, *Pm3b,* and *Pm3d* were present in the field. In 2015, we placed pots in the field containing winter wheat cultivar Michigan Amber/8*CC which contains the endogenous *Pm3f* allele. The potted plants showed powdery mildew infections (data not shown), and thus, *Bgt* strains virulent on *Pm3f* were present in the field as well.

### The four allele-pyramided lines are more powdery mildew resistant in the field than their parental lines carrying a single *Pm3* allele

Line Pm3a had AUDPC scores of 179 and 99 in 2015 and 2016, respectively (Fig. 1a, b). Line Pm3d had similar AUDPC scores as line Pm3a in both years (Fig. 1a, b). Line Pm3b had AUDPC scores of 99 and 16 in 2015 and 2016, respectively (Fig. 1a, b). Line Pm3f was completely powdery mildew resistant in both years with AUDPC scores of 0 (Fig. 1a, b). Thus, the transgenic allele *Pm3f* provided the best, *Pm3b* an intermediate and alleles *Pm3a* and *Pm3d* the weakest disease resistance. In agreement with this observation, pyramided line Pm3a × Pm3d was the least powdery mildew resistant among the four pyramided lines in both years (Fig. 1a, b). In 2015, it had an AUDPC score of 104, which still was lower than the scores of their parental lines (Pm3d: AUDPC score 149 and Pm3a: AUDPC score 179) (Fig. 1a). In 2016, pyramided line Pm3a × Pm3d had an AUDPC score of 35, which as well was lower than the score of their parental lines (Pm3a: AUDPC score 99 and Pm3d: AUDPC score 104) (Fig. 1b). Pyramided line Pm3b × Pm3d had AUDPC scores of 22 in 2015 and 4 in 2016, respectively, and thus showed only trace infections and was more resistant than its parental lines Pm3b and Pm3d (Fig. 1a, b). The two pyramided lines Pm3a × Pm3b and Pm3b × Pm3f in both years were completely powdery mildew resistant with AUDPC scores of 0 (Fig. 1a, b). Taken together, these results show an improved powdery mildew resistance in the pyramided lines compared to their parental lines.

### Agronomic traits of the pyramided lines are similar to the mean of the corresponding parental lines

**Fig. 2:**
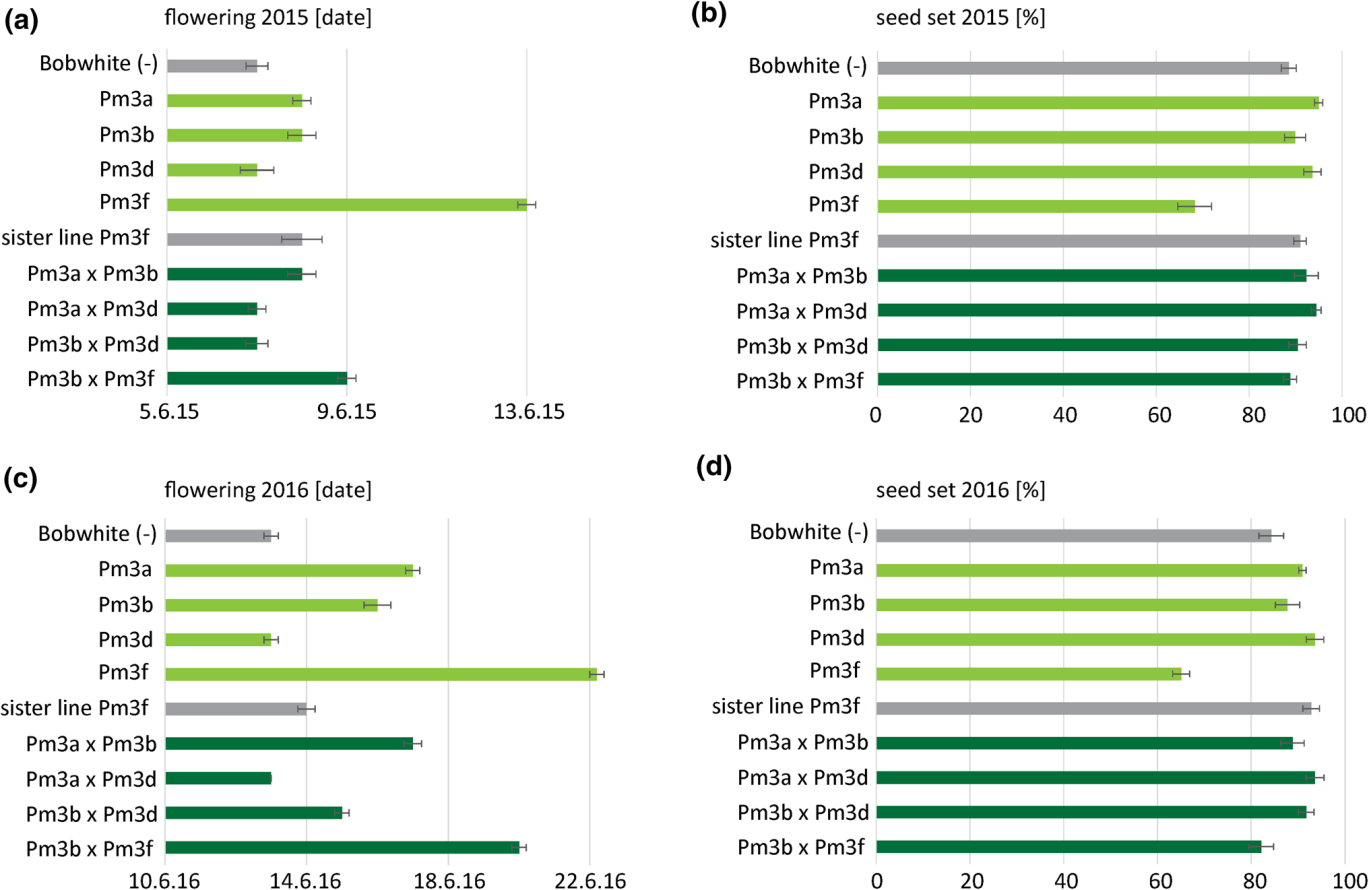
Flowering date (**a**, **c**) and seed set rate (**b**, **d**) of pyramided lines (dark green), parental lines (light green), non-transformed Bobwhite (grey), and sister line Pm3f (grey) are shown for plants grown in field season 2015 (**a**, **b**) and 2016 (**c**, **d**). Standard errors of five replicates (five microplots per line) are indicated (color figure online)

To assess potential pleiotropic effects on plant development caused by pyramiding of *Pm3* alleles, we scored flowering dates and seed set rates of the field grown lines. The summer 2015 was above-average sunny, warm, and dry, and overall, the plants developed well. In 2016, the weather conditions were not conducive to wheat growth. It was mostly rainy and moist, and the plants were stressed. Thus, negative effects on plant development were more distinct in 2016 compared to 2015; however, the overall plant development patterns were similar in both years (Fig. 2). In 2015, non-transformed Bobwhite, parental lines Pm3a, Pm3b, and Pm3d as well as sister line Pm3f and the pyramided lines Pm3a × Pm3b, Pm3a × Pm3d, and Pm3b × Pm3d all flowered within the same 2 days, whereas line Pm3f flowered almost 1 week later (Fig. 2a). Interestingly, pyramided line Pm3b × Pm3f only flowered 2 days later than non-transformed Bobwhite, and thus, the late flowering phenotype of line Pm3f was partly restored in the progeny line Pm3b × Pm3f (Fig. 2a). In 2015, line Pm3f showed a low seed set rate of 68% compared to the seed set rates of 89–95% of all the other lines, including pyramided line Pm3b × Pm3f (Fig. 2b). In 2016, non-transformed Bobwhite, parental line Pm3d, sister line Pm3f, and pyramided line Pm3a × Pm3d all flowered within the same 2 days (Fig. 2c). Pyramided line Pm3b × Pm3d flowered 2 days later than non-transformed Bobwhite and parental lines Pm3b and Pm3a flowered 3 and 4 days later than non-transformed Bobwhite, respectively (Fig. 2c). Pyramided line Pm3a × Pm3b flowered 4 days later than non-transformed Bobwhite as well (Fig. 2c). Parental line Pm3f flowered 9 days later than non-transformed Bobwhite (Fig. 2c). Again, this late flowering phenotype was partly restored in progeny pyramided line Pm3b × Pm3f, which flowered 7 days after non-transformed Bobwhite (Fig. 2c). In 2016, line Pm3f had a low seed set rate of 65% compared to all the other lines with seed set rates of 82–94% (Fig. 2d). The low seed set rate of Pm3f was largely restored in progeny line Pm3b × Pm3f with a rate of 82% (Fig. 2d).

### Allele-pyramided lines are more powdery mildew resistant than the corresponding line mixtures in the yield plots; however, the pyramided lines and the corresponding line mixtures have similar yields

**Fig. 3:**
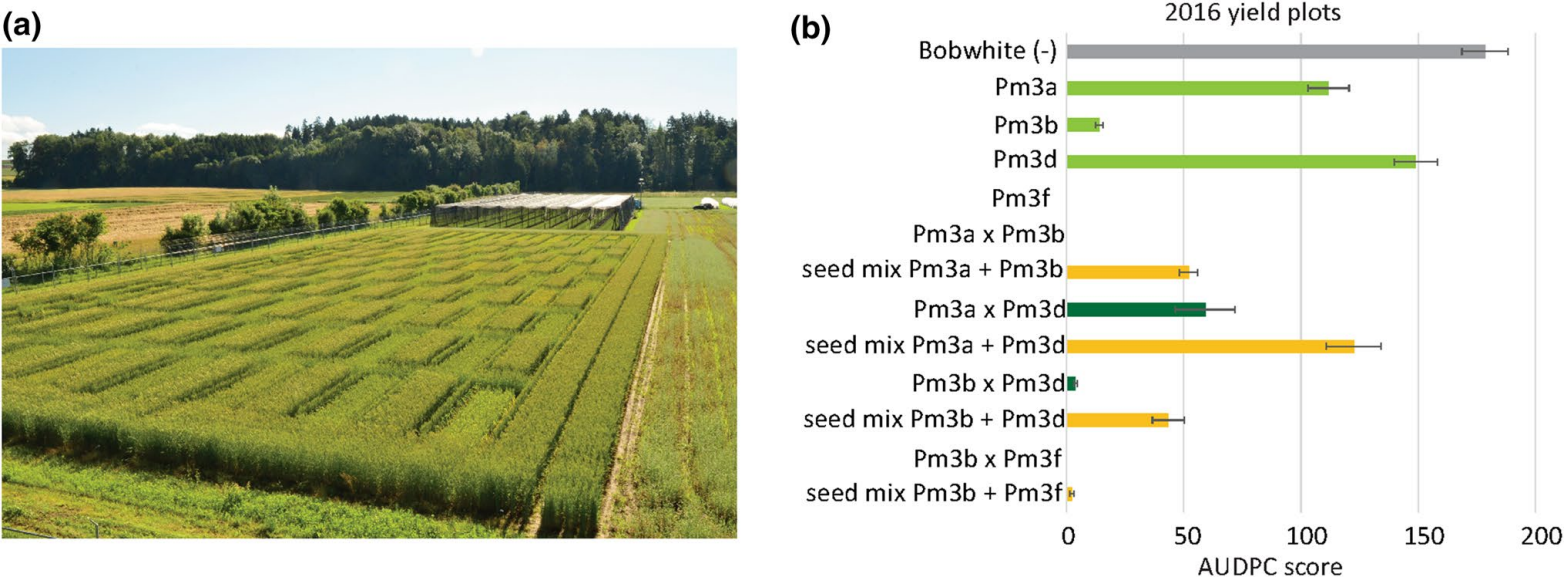
**a** Photograph of field trial 2016. The yield plots are arranged in a chessboard-like design, bordered on all sides by plots of powdery mildew-resistant triticale cultivar Trado. On the left border of the field, the three rows containing the smaller so-called microplots are visible. **b** Powdery mildew infection in yield plots of pyramided lines (dark green), line mixtures (yellow), components of line mixtures in pure stand/parental lines (light green), and non-transformed Bobwhite (grey). Mean of area under disease progress curve (AUDPC) scores was calculated of four independent replicates (four yield plots) per line grown in field season 2016 (color figure online)

**Fig. 4:**
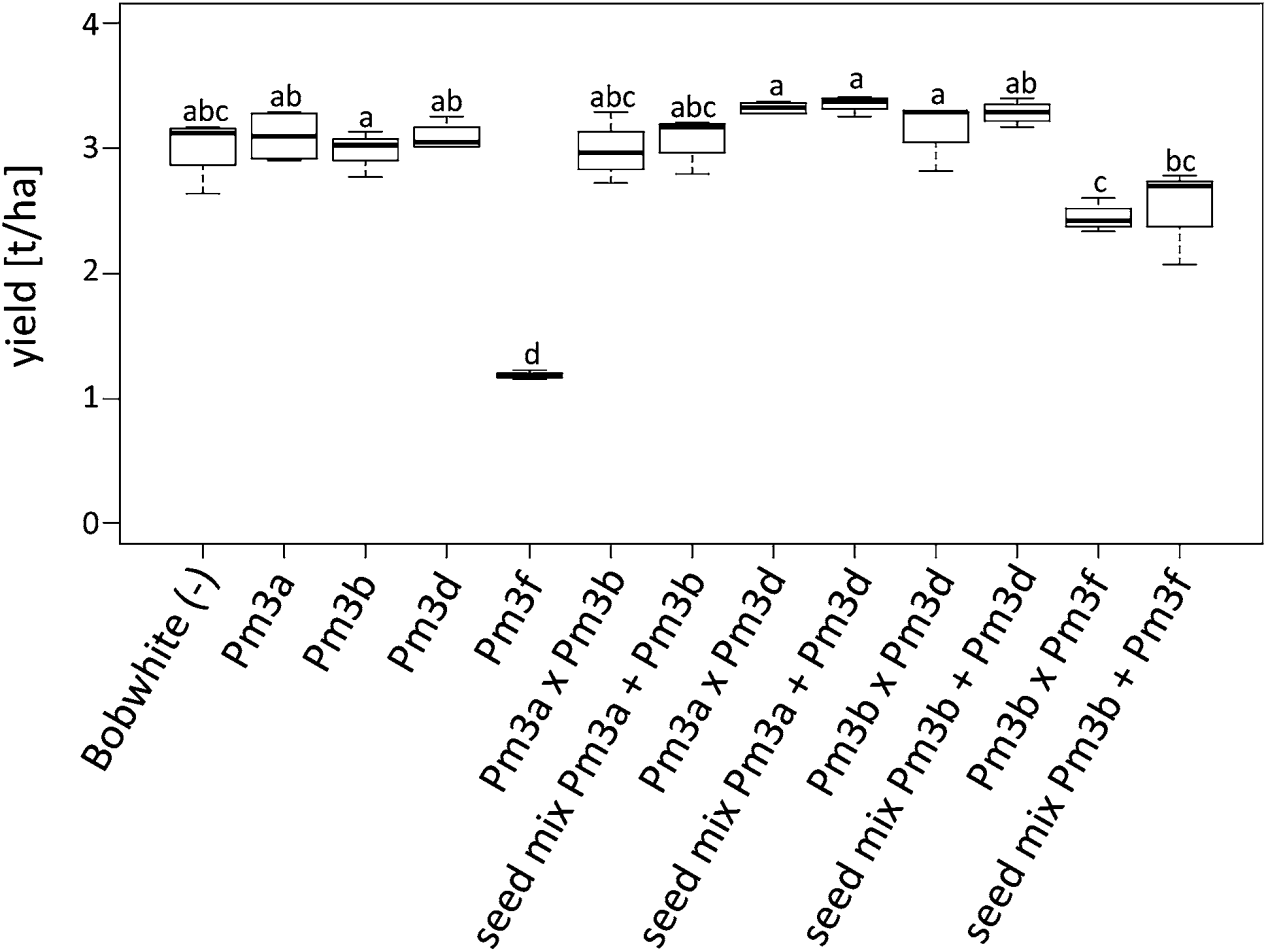
Yields of pyramided lines, line mixtures, components of line mixtures in pure stand/parental lines and non-transformed Bobwhite grown in yield plots in 2016. Medians are indicated of four independent replicates (four yield plots) per line. Same letters indicate no significant difference in pairwise comparisons

In 2016, in addition to the microplots, we grew all the parental lines, the pyramided lines, and the corresponding line mixtures in larger yield plots (Fig. 3a). Line mixtures consisted of a 1:1 ratio seed mixture of the parental lines in the following four combinations: Pm3a + Pm3b, Pm3a + Pm3d, Pm3b + Pm3d, and Pm3b + Pm3f. Overall, the powdery mildew AUDPC scores of the lines grown in the yield plots (Fig. 3b) were similar to the AUDPC score of the lines grown in the microplots (Fig. 1). The line mixtures had similar AUDPC scores compared to the mean of the AUDPC scores of the corresponding lines in pure stands (Fig. 3b). Compared to the corresponding pyramided lines, all four line mixtures had higher AUDPC scores, and thus, the pyramided lines were more powdery mildew resistant than the corresponding line mixtures (Fig. 3b). In terms of yield, there was no significant difference between the pyramided lines and the corresponding line mixtures (Fig. 4). Overall, the parental lines, the pyramided lines, and the line mixtures had similar yield, except for lines and mixtures containing *Pm3f* (Fig. 4). Parental line Pm3f had a significantly lower yield than all the other lines (Fig. 4). The severe yield loss phenotype of line Pm3f was partly restored in progeny pyramided line Pm3b × Pm3f, which had a similar yield as line mixture Pm3b + Pm3f (Fig. 4).

### Transgene expression levels in pyramided lines are additive

**Fig. 5:**
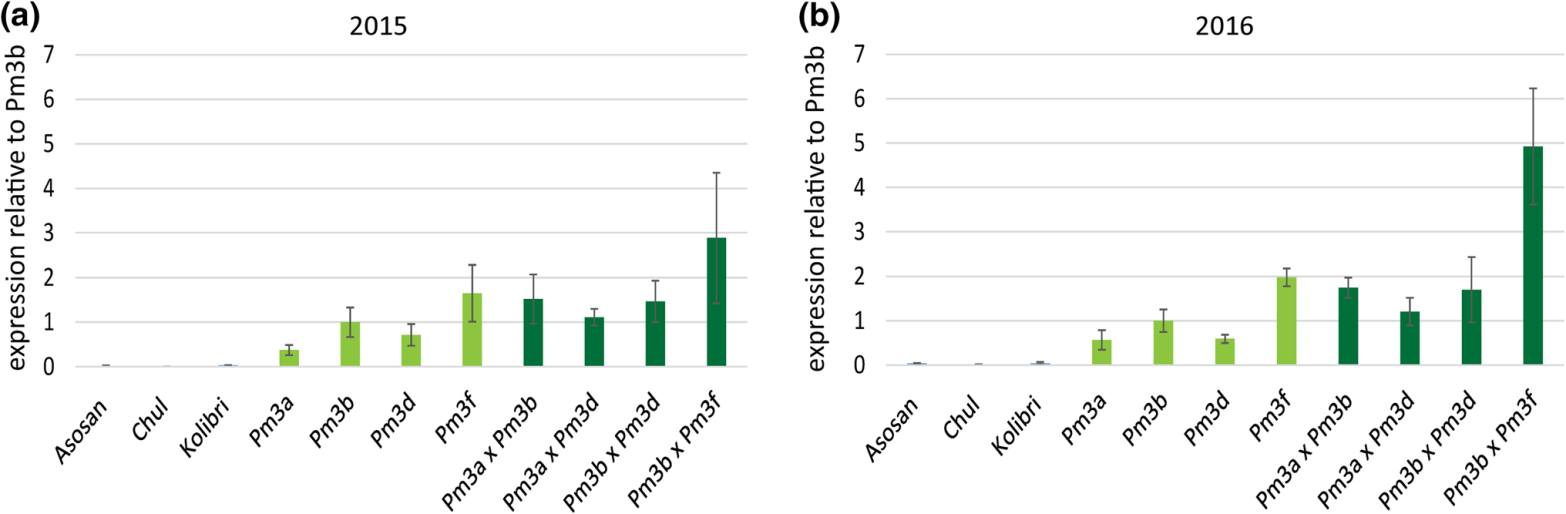
*Pm3* expression levels determined by RT qPCR using conserved probes (i.e., no distinction between *Pm3* alleles) of the field grown pyramided lines (dark green), parental lines (light green), and cultivars Asosan, Chul, and Kolibri (grey) carrying the endogenous alleles *Pm3a*, *Pm3b,* and *Pm3d*, respectively. Expression levels were calculated relative to expression level of *Pm3b* from field grown line Pm3b. Mean and standard deviation of five independent replicates (pooled flag leaf samples from three plants per plot, from five microplots) are indicated of lines and cultivars grown in 2015 (**a**) and 2016 (**b**) (color figure online)

**Fig. 6:**
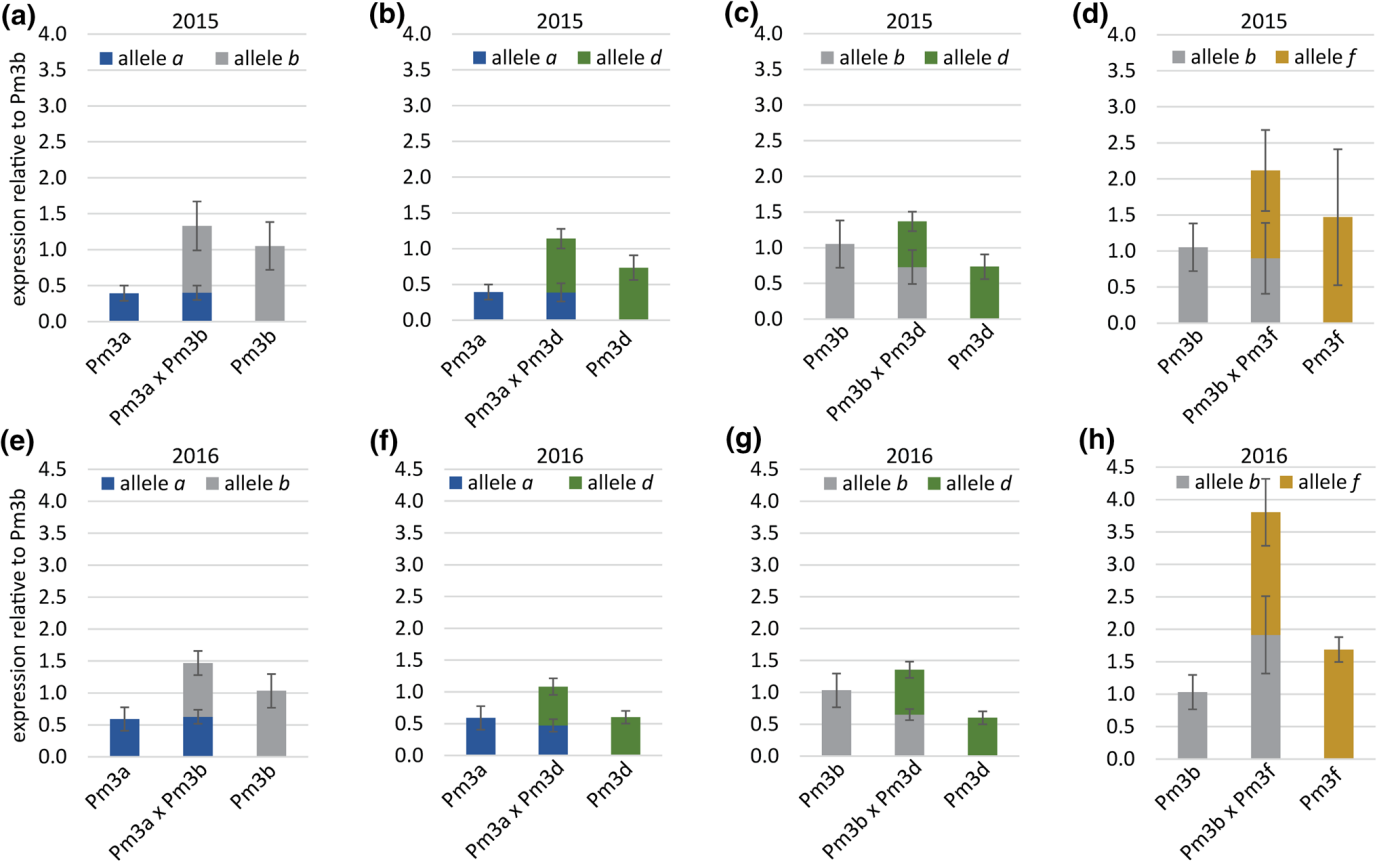
*Pm3* expression levels determined by RT qPCR using allele-specific probes. Expression levels are calculated relative to expression level of *Pm3b* from field grown line Pm3b. Mean and standard deviation of five independent replicates (pooled flag leaf samples from three plants per plot, from five plots) are indicated of lines grown in field season 2015 (**a**–**d**) and 2016 (**e**–**h**)

We measured transgene expression levels of flag leaf samples from the field grown cultivars and lines using non-allele-specific probes in RT qPCR assays. We calculated expression levels relative to *Pm3b* from line Pm3b as reference, because this line was already used as reference in earlier studies and has an intermediate level of transgene expression (Brunner et al. 2011, 2012). In both years, cultivars Asosan, Chul, and Kolibri carrying the endogenous *Pm3* alleles showed very low expression levels (2015: 0.02, 0.01, and 0.03, respectively; 2016: 0.04, 0.01, and 0.04, respectively) compared to line Pm3b (Fig. 5). This is in agreement with the previous results (Brunner et al. 2011, 2012). In both years, parental lines Pm3a and Pm3d showed slightly lower transgene expression levels than line Pm3b with relative scores of 0.38 (Pm3a) and 0.72 (Pm3d) in 2015 and 0.57 (Pm3a) and 0.59 (Pm3d) in 2016 (Fig. 5). In both years, parental line Pm3f showed high transgene expression levels with relative scores of 1.65 in 2015 and 1.98 in 2016 (Fig. 5). All four pyramided lines showed additive total transgene expression levels, i.e., transgene expression levels were the sum of the transgene expression levels of the parental lines (Fig. 5). No suppression among *Pm3* alleles at the transcriptional level was observable in any of the pyramided lines (Fig. 5). We then designed allele-specific RT qPCR probes to determine the contribution to transgene expression levels from each of the two *Pm3* alleles in the pyramided lines. Overall, the transgene expression levels using the allele-specific probes (Fig. 6) were similar to the data which we obtained using the non-allele-specific probes (Fig. 5). The allele-specific probes revealed that the transgene expression levels of the individual alleles in the pyramided lines were similar to the transgene expression levels of the individual alleles in the parental lines (Fig. 6), resulting in additive transgene expression levels.

## Discussion

### Combination of allele specificities partially explains improved powdery mildew resistance in pyramided lines

All four pyramided lines were more powdery mildew resistant than their corresponding parental lines in both field seasons (Fig. 1). There are two explanations for resistance, one is related to characteristics of the used plant genotypes and the other is related to characteristics of the pathogen population. In 2015 and 2016, the *Bgt* population present at the site of the field trial consisted at least partially of strains virulent on all four alleles (*Pm3a, b, d,* and *f*) present in the pyramided lines (Fig. 1). Possibly, *Bgt* strains virulent on *Pm3a* and *Pm3d* were more abundant at the site of the field trial than strains virulent on *Pm3b* and *Pm3f*. This would partially explain the higher powdery mildew infections on parental lines Pm3a and Pm3d as well as on the pyramided line Pm3a × Pm3d. Earlier studies on virulence frequency of Swiss powdery mildew races to *Pm3* alleles present at the field site at Zurich-Reckenholz in 2007 showed that *Bgt* strains virulent on *Pm3f* (73% virulence frequency) were more frequent than *Bgt* strains virulent on *Pm3a* (6%), *Pm3b* (4%), and *Pm3d* (10%) (Brunner et al. 2012). Our results suggest a shift in the *Bgt* population since 2007, resulting in a much lower frequency of *Bgt* strains virulent on *Pm3f*. However, the high powdery mildew resistance of line Pm3f is probably due to other factors than the pathogen population composition, because cultivar Michigan Amber/8*CC which contains the endogenous *Pm3f* allele, showed powdery mildew infection.

Much has been reported about additive (Hu et al. 2012; Kim et al. 2012; Liu et al. 2000; Xiao et al. 2017) or negative (Chen et al. 2013; Knott 2000; Liu et al. 2013; McIntosh et al. 2011) gene action in various plant species with resistance gene combinations. In these studies, resistance gene combination was achieved by classical crossbreeding and marker-assisted selection or in the case of Chen et al. (2013) by generation of interspecific crosses between tetraploid wheat and *Ae. tauschii*. Additive or negative gene action has also been reported from resistance gene combinations achieved by genetic engineering resulting in trans- or cisgenic plants. Additive gene action was reported for transgenic flax with pyramided rust resistance alleles (Chen et al. 2008) and for transgenic potatoes with pyramided late blight-resistance genes (Zhu et al. 2012; Jo et al. 2014), and suppression was reported from transgenic wheat carrying *Pm3* and *Pm8* (Hurni et al. 2014). However, no field trials were performed with the transgenic flax lines or the transgenic wheat lines carrying *Pm3* and *Pm8*. Tundo et al. (2016) pyramided various genes encoding different inhibitors to generate transgenic wheat resistant to *Fusarium graminearum*. Some inhibitor combinations improved the resistance spectrum (Tundo et al. 2016). In the case of potatoes with improved resistance spectra against *Phytophthora infestans,* both classical crossbreeding with marker-assisted selection and genetic engineering have been used and field trials were conducted. The two resistance genes *R*_*Pi*-*mcd1*_ and *R*_*Pi*-*ber*_ were combined by crossbreeding and field trials revealed an additive effect of the two genes (Tan et al. 2010). Furthermore, potato differential lines Ma*R8* and Ma*R9* show broad spectrum late blight resistance due to multiple pyramided *R* genes (Kim et al. 2012). Zhu et al. (2012) transformed potato cultivar Desiree simultaneously with three broad spectrum potato *R* genes which resulted in a resistance spectrum which was the sum of the spectra from the three individual *R* genes (Zhu et al. 2012). Jo et al. (2014) then developed a marker-free transformation procedure to obtain cisgenic potato lines expressing several pyramided *R* genes (Jo et al. 2014). The cisgenic potato lines showed improved resistance in field trials (Haverkort et al. 2016; Haesaert et al. 2015). Thus, in potato, the pyramiding of *R* genes is a successful approach to broaden the resistance spectrum, as seems to be the case for our *Pm3* allele-pyramided wheat lines.

### Resistance suppression among pyramided *Pm3* alleles observed in laboratory experiments was not confirmed in the field

Stirnweis et al. (2014) tested powdery mildew resistance of the four pyramided lines used in this study in infection tests in the laboratory on leaf segments of 10-day-old seedlings. When *Bgt* isolates 97011 and 98229 which were used for infection, a resistance suppression was observed, namely Pm3 variants Pm3b and Pm3c suppressed the function of both Pm3 variants Pm3a and Pm3f. Surprisingly, we did not observe functional suppression of Pm3 variants Pm3a or Pm3f by variant Pm3b in the field grown plants; on the contrary, pyramided lines Pm3a × Pm3b and Pm3b × Pm3f were completely powdery mildew resistant in both years with AUDPC scores of 0 (Fig. 1). Stirnweis et al. (2014) observed the suppression phenotype of the allele-pyramided lines only when using *Bgt* isolates 97011 (avirulent on *Pm3a/d/f*, virulent on *Pm3b/c*) and 98229 (avirulent on *Pm3a/d/f*, virulent on *Pm3b/c*) but not when using isolates *Bgt* 07201 (avirulent on *Pm3b*, virulent on *Pm3a/f*) and 07230 (avirulent on *Pm3b/c*, virulent on *Pm3a/f*). The infection tests were performed on 10-day-old wheat seedlings. The disparate results from the seedling tests and the field trials cannot be attributed to the age difference of the plants, because it is known that *Pm3* already is effective at the 10-day-old seedling stage (Brunner et al. 2010, 2011, 2012; Stirnweis et al. 2014). Stirnweis et al. (2014) further investigated the suppression mechanism and concluded it occurred at the post-translational level, because RNA and protein levels of Pm3 alleles/variants were unaffected by the presence of suppressing Pm3 alleles/variants. In transiently transformed *N. benthamiana* leaves expressing *Pm3b* and autoactive *Pm3f*-*D501V,* Stirnweis et al. (2014) observed suppression of the hypersensitive response, which suggests that in the presence of activated Pm3f, the suppression is independent of the presence of *Bgt* isolates. Our result of the absence of observable allele suppression in the same pyramided lines when grown in field conditions adds to the puzzle of the underlying mechanism of Pm3 activation and function. Further molecular studies of laboratory and field grown plants infected with various *Bgt* isolates are necessary to elucidate the observed resistance or suppression phenotypes. At this point, we only can speculate on possible factors explaining the disparate results obtained in laboratory and field trials. One factor, besides the *Bgt* population composition, could be a difference in immune system activity of laboratory and field grown plants; for instance, an avirulent *Bgt* isolate landing on a field grown plant could prime the plant for improved immunity and subsequent *Bgt* strains virulent under laboratory conditions could remain avirulent under field conditions. In our field trial, besides the naturally occurring *Bgt* population, *Bgt* isolate 96224, which is avirulent on all field tested *Pm3* alleles, was present in the field because of the *Bgt* 96224 infected spreader rows.

### Positive correlation between total transgene expression level and powdery mildew resistance in transgenic wheat lines of cultivar Bobwhite

In the field grown parental and pyramided lines, all in the background of cultivar Bobwhite, we observed a positive correlation between total transgene expression level and level of powdery mildew resistance. The three lines Pm3b × Pm3f, Pm3a × Pm3b, and Pm3f had the highest total transgene expression levels in both years and where completely powdery mildew resistant in both years, whereas lines Pm3a and Pm3d were quite susceptible in both years and showed the lowest transgene expression levels in both years (Figs. 1, 5). Only few studies so far investigate the correlation between field resistance and transgene expression levels. Han et al. (2012) reported a positive correlation of the level of transgene expression (cDNA of bovine lactoferrin) and resistance of wheat to *F. graminearum* (Han et al. 2012). Krattinger et al. (2016) reported a positive correlation between level of transgene expression of resistance gene *Lr34* in rice and level of leaf tip necrosis, but transgene expression level was not positively correlated to disease resistance (Krattinger et al. 2016). Both these studies did not conduct field trials with the transgenic plants. High levels of transgene expression in plants can lead to gene silencing (Pickford and Cogoni 2003). However, we did not observe gene silencing in any of our field tested lines. Kim et al. (2012) reported a background dependence of resistance gene action in potato. In our study, allele *Pm3d* performed better in the endogenous background of cultivar Kolibri than in our Bobwhite *Pm3d* overexpressing line, even though *Pm3d* was much higher expressed in Bobwhite line Pm3d than in Kolibri (Fig. 5). Cultivar Kolibri has been used agronomically in Switzerland since 1975. In contrast to cultivar Bobwhite, Kolibri is locally adapted and thus is likely to carry additional, unknown powdery mildew resistance factors (e.g., minor, quantitative resistance genes), which are absent in cultivar Bobwhite. Thus, even though we observed a positive correlation between transgene expression level and powdery mildew resistance in transformed cultivar Bobwhite, *Pm3* expression levels alone do not explain strength of powdery mildew resistance.

### *Pm3* allele pyramiding is a successful approach for enhancing powdery mildew field resistance

Overactive immunity in plants can have negative effects on plant development (Belkhadir et al. 2014; Lozano-Durán and Zipfel 2015). We did not observe a correlation of total transgene expression level and abnormal plant development. On the contrary, pyramided line Pm3b × Pm3f, which had a higher total transgene expression level than parental line Pm3f, showed yield, flowering date, and seed set rate more similar to non-transformed Bobwhite than to parental line Pm3f, which had an abnormal plant development phenotype (Figs. 2, 4, 5, 6). The observed severely delayed flowering and low seed set rate of line Pm3f, but not of sister line Pm3f, is in agreement with Brunner et al. (2012). In the case of pyramided line Pm3b × Pm3f, crossbreeding of “abnormal” line Pm3f with line Pm3b to a large extent restored normal plant development. The other three pyramided lines did not show any negative effects of allele pyramiding on plant development neither. *Pm3* allele pyramiding did not have a negative effect on the plants and was a successful approach in enhancing field resistance to powdery mildew. Furthermore, it was a more successful approach than using line mixtures (Fig. 3b). Brunner et al. (2012) showed improved powdery mildew resistance of the multilines compared to the lines in pure stands. In our study, we showed further improvement of resistance by allele pyramiding, which, in the two pyramided lines Pm3a × Pm3b and Pm3b × Pm3f, resulted in plants completely free of powdery mildew infection. We attribute the improved resistance to the two effects of enhanced total transgene expression levels and allele-specificity combinations. The transgenic lines used in this study could be further improved with characteristics of interest by classical crossbreeding.

#### **Author contribution statement**

SB, SH, BK, and TK designed and carried out the field trial; GH designed and carried out the RT qPCR experiments; TK, SB, and BK wrote the manuscript.
